# Supplementary material for: Hemophagocytic Lymphohistiocytosis Associated with Synergistic Defects of AP3B1 and ATM Genes: A Case Report and Literature Review
Source: J Clin Med. 2022 Dec 22;12(1):95. doi: 10.3390/jcm12010095 (PMC9821123; doi:10.3390/jcm12010095)
Supplement: Supplementary file 1 [file jcm-12-00095-s001.zip › Additional files/primer/AP3B1 and ATM gene Primer.docx]

| Genes | Forward primers(5'-3') | Reverse primers(5'-3') |
| --- | --- | --- |
| AP3B1 | ATGAAAGGCAGCAGTAGTGG | GAAGAATGCTTGGCTTTGGG |
| ATM | CACACCCGGCCTAAAGTTGT | GTCTCCTCTCCTTGCCATCG |
