# Supplementary figures and images for: Hemophagocytic Lymphohistiocytosis Associated with Synergistic Defects of AP3B1 and ATM Genes: A Case Report and Literature Review
Source: J Clin Med. 2022 Dec 22;12(1):95. doi: 10.3390/jcm12010095 (PMC9821123; doi:10.3390/jcm12010095)

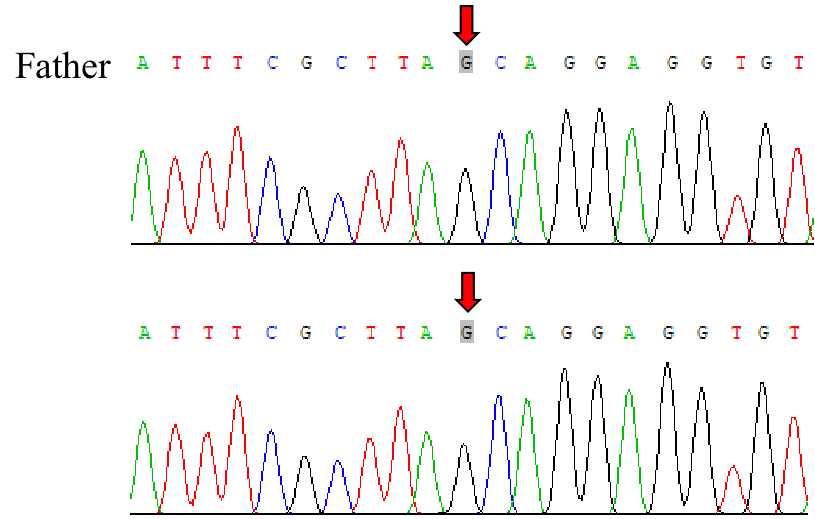

Supplement: Supplementary file 1 [file jcm-12-00095-s001.zip › supplementary figure S1.jpg]

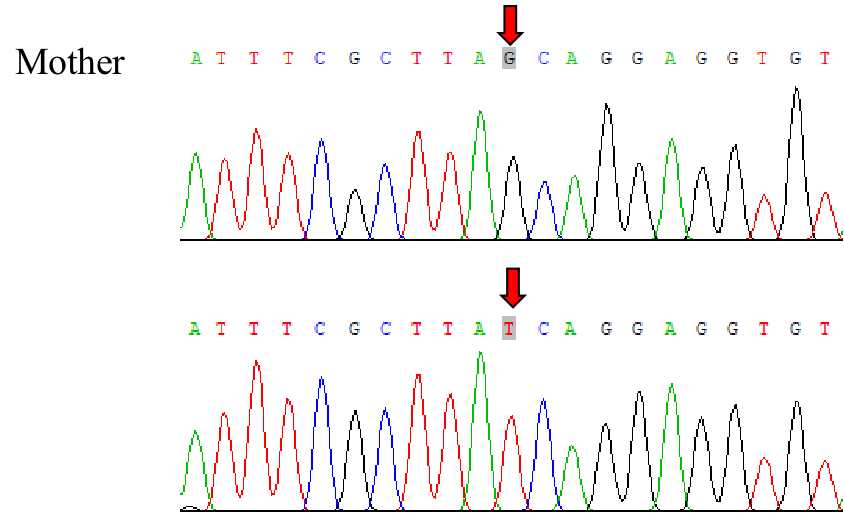

Supplement: Supplementary file 1 [file jcm-12-00095-s001.zip › supplementary figure S2.jpg]

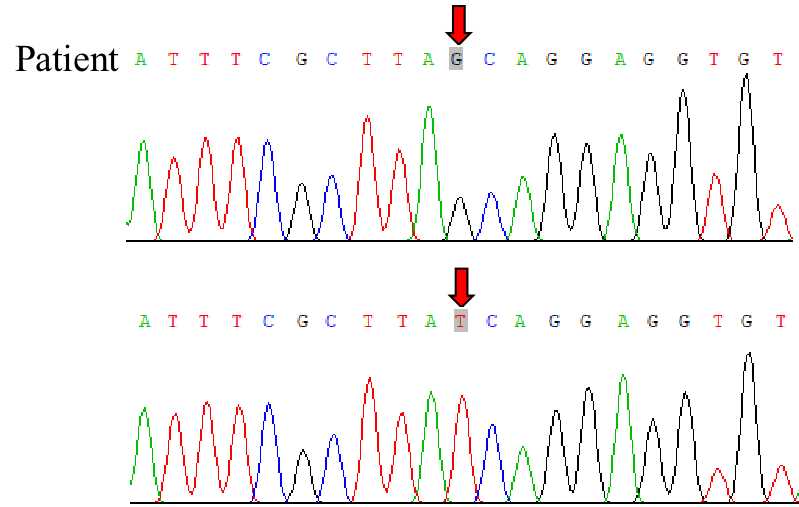

Supplement: Supplementary file 1 [file jcm-12-00095-s001.zip › supplementary figure S3.jpg]
